# Supplementary material for: Protein Kinase C δ: a critical hub regulating macrophage immunomodulatory functions during Mycobacterium tuberculosis infection
Source: bioRxiv. 2025 May 23:2025.05.19.653976. Preprint. [Version 1] doi: 10.1101/2025.05.19.653976 (PMC12139767; doi:10.1101/2025.05.19.653976)
Supplement: Supplement 1 [file media-1.pdf]

Supplementary Fig 1.

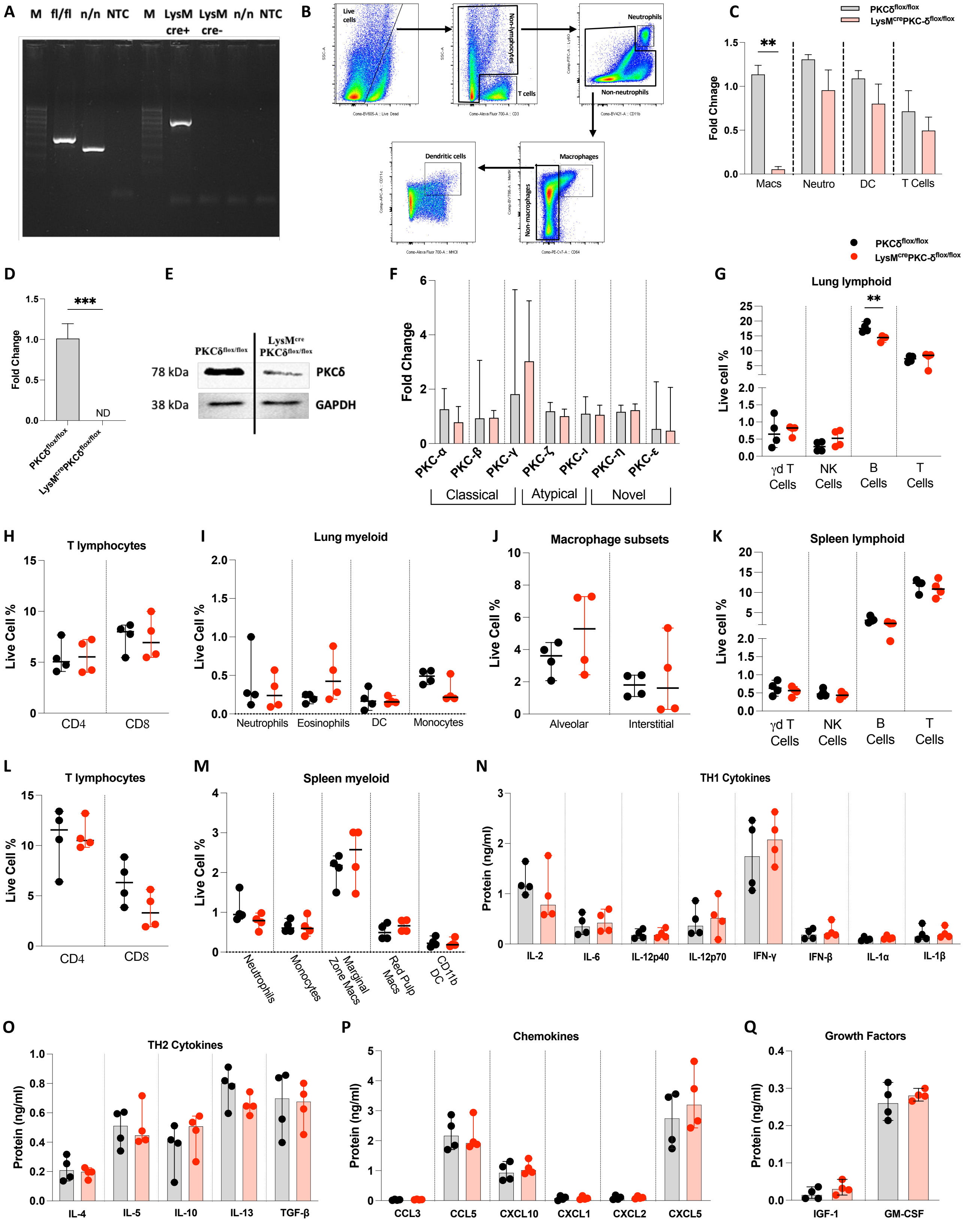

Supplementary Figure 1. LysM^{cre}PKCδ$^{flox/flox}$ mice are indistinguishable from PKCδ$^{flox/flox}$ littermate control at the naive state. [A] Integrity of loxP-floxed PKCδ and presence of LysMcre was confirmed in the genomic DNA extracted from the tail cuts of PKCδ$^{flox/flox}$ and LysM^{cre}PKCδ$^{flox/flox}$ mice. Representative agarose gel demonstrates the marker (m), wild-type (n/n), floxed PKCδ (fl/fl), LysM^{cre}PKCδ$^{flox/flox}$ (LysMcre+), PKCδ$^{flox/flox}$ (LysMcre-), non-template control (NTC). **[B]** Gating strategy for lung sorted immune cells and confirmation of PKCδ deletion in macrophages by qRT-PCR. **[C]** PKCδ expression was determined in sorted immune cell populations by qRT-PCR. Expression levels are normalized to the endogenous housekeeping gene Hprt. **[D-E]** Deletion of PKCδ in bone- marrow derived macrophages was confirmed by qRT-PCR and western blot analysis. HPRT and GAPDH were used as a normalizing control in qRT-PCR and Western blot respectively. **[F]** Protein kinase C isoform-specific primers were used for gene expression analysis in PKCδ deficient bone-marrow derived macrophages (LysM^{cre}PKCδ$^{flox/flox}$) as compared to the control (PKCδ$^{flox/flox}$) by qRT-PCR. **[G-M]** Various lymphoid and myeloid immune populations were detected by flow cytometry in the lung [G-J] and spleen [K-M] in both PKCδ$^{flox/flox}$ and LysM^{cre}PKCδ$^{flox/flox}$ mice. **[N-Q]** Determining cytokine levels in lung homogenates collected from PKCδ$^{flox/flox}$ and LysM^{cre}PKCδ$^{flox/flox}$ mice. All data shown mean+/- SD and is representative of two independent experiments with n=4 mice/group. Statistical analyses were performed using an unpaired student t-test. Asterisks are defining significance compared to the control group as: $^{**}p < 0.01$, $^{***}p < 0.001$.

Supplementary Fig 2.

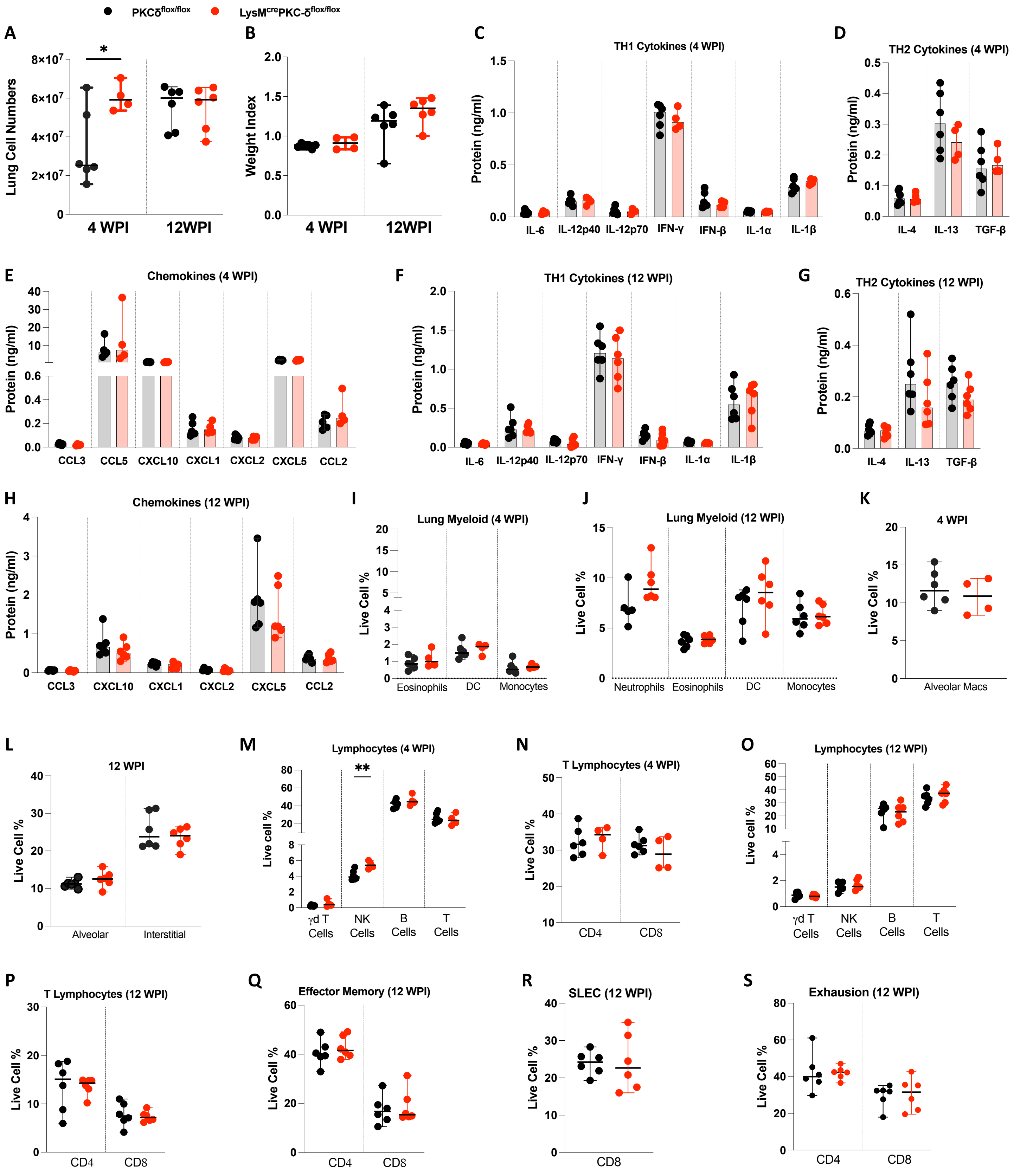

Supplementary Figure 2. Lung cell numbers, cytokine profiling, and immune cell phenotyping of LysM^{cre}PKCδ$^{flox/flox}$ mice during acute (4WPI) and chronic (12 WPI) *Mtb* infection. [A] Lung cell numbers and **[B]** weight index were determined at acute and chronic stage of *Mtb* infection. **[C-H]** Determining cytokine and chemokine levels in lung homogenates collected from PKCδ$^{flox/flox}$ and LysM^{cre}PKCδ$^{flox/flox}$ mice at acute and chronic stage of *Mtb* infection. **[I-S]** Various myeloid and lymphoid immune cell populations were detected by flow cytometry at acute and chronic stage of *Mtb* infection. All data shown mean+/- SD and is representative of two independent experiments with n=4-6 mice/group. Statistical analyses were performed using an unpaired student t-test. Asterisks are defining significance compared to the control group as: *$p < 0.05$, **$p < 0.01$.

Supplementary Fig 3.

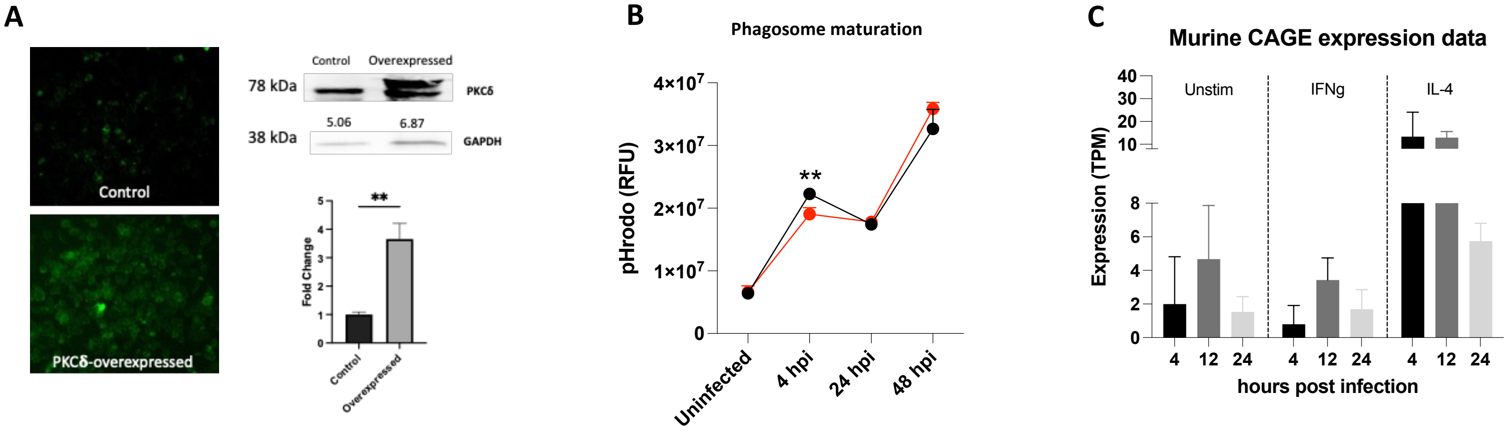

Supplementary Figure.3. PKCδ overexpression in RAW264.7 murine macrophages, determination of phagosome maturation in LysM^{cre}PKCδ$^{flox/flox}$ BMDMs and murine PKCδ expression during *Mtb* infection (adapted from FANTOM5 CAGE database). [A] Lentivirus-mediated overexpression of PKCδ in RAW264.7 murine macrophage cell line confirmed by ZOE fluorescence imaging, western blot, and qRT-PCR. **[B]** Determination of phagosome maturation (Relative fluorescence unit) based on pH change in the phagolysosomal compartment in bone-marrow-derived macrophages from LysM^{cre}PKCδ$^{flox/flox}$ and PKCδ$^{flox/flox}$ mice. **[C]** Determining PKCδ expression in classically (IFNg-stimulated) or alternatively (IL-4 stimulated) activated murine macrophages utilizing publicly available FANTOM5 CAGE database. Asterisks are defining significance compared to the control group as: **$p < 0.01$.

Supplementary Fig 4.

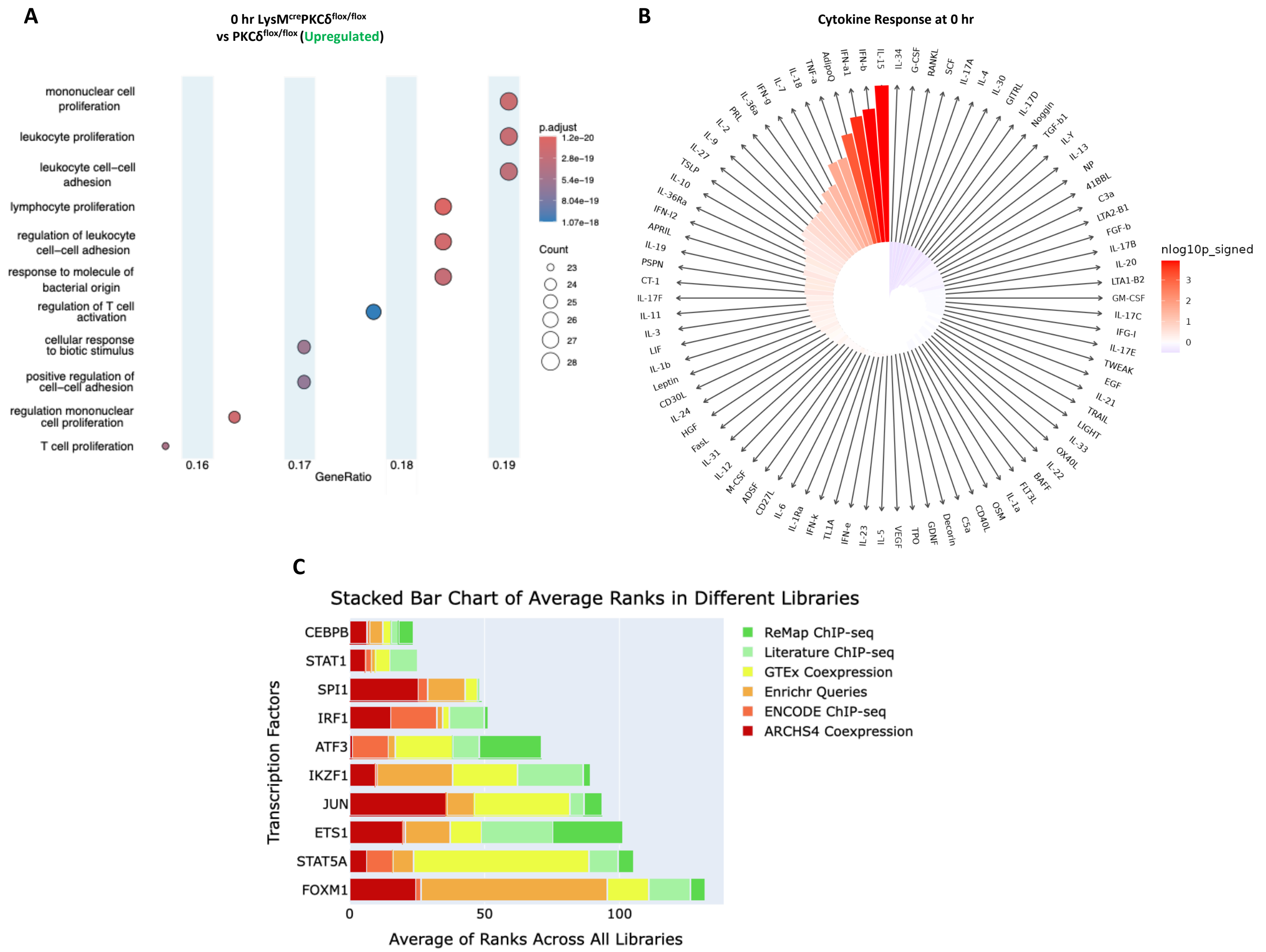

Supplementary Figure.4. Gene ontology, cytokine response (IREA), and ChEA analysis in the LysM^{cre}PKCδ$^{flox/flox}$ BMDMs at naive state. [A] Horizontal dot plots (Ora) detailing the association of enriched Gene Ontology (GO) biological processes are shown between LysM^{cre}PKCδ$^{flox/flox}$ and PKCδ$^{flox/flox}$ BMDMs at 0 hr (uninfected) time point. **[B]** IREA cytokine enrichment plot showing the enrichment score (ES) for each of the cytokine response in LysM^{cre}PKCδ$^{flox/flox}$ BMDMs at 0 hr (uninfected) time point. Bar length is representing the ES with darker red (enriched in LysM^{cre}PKCδ$^{flox/flox}$ BMDMs) and darker blue (enriched in PKCδ$^{flox/flox}$ BMDMs). **[C]** Horizontal bar chart representing the top ranked transcription factors at 0 hr (uninfected) time point according to their average integrated scores across all the libraries. All data shown are analysed and produced using R studio packages and appyters web-based software.

Supplementary Fig 5.

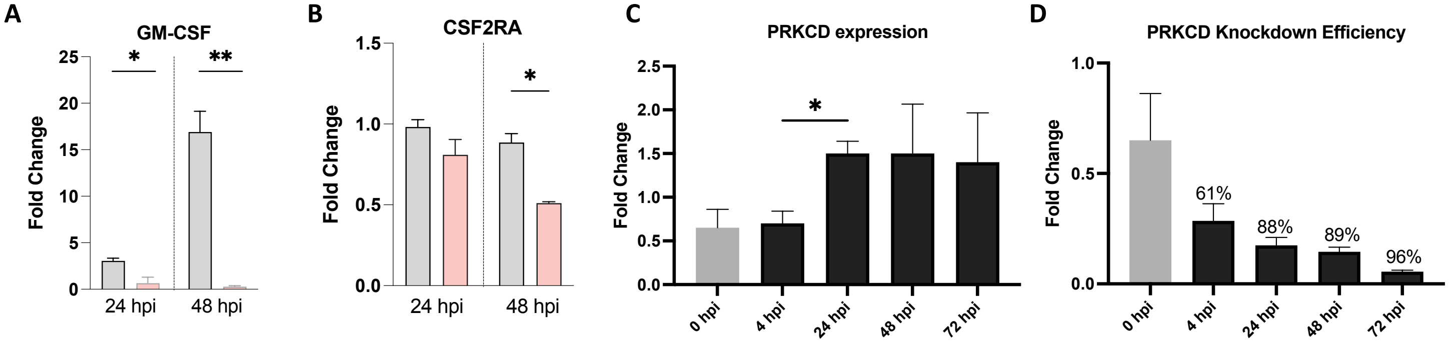

Supplementary Figure.5. mRNA expression of GM-CSF and CSF2RA in LysM^{cre}PKCδ$^{flox/flox}$ BMDMs, mRNA expression of PKCδ in MDMs and siRNA mediated PKCδ knockdown efficiency in MDMs during *Mtb* infection. [A-B] mRNA expression of GM-CSF and CSF2RA in bone-marrow-derived macrophages from LysM^{cre}PKCδ$^{flox/flox}$ and PKCδ$^{flox/flox}$ mice during *Mtb* infection at indicated time points. **[C]** mRNA expression of PRKCD in human monocyte-derived macrophages at indicated time points during *Mtb* infection. **[D]** siRNA mediated knockdown efficiency of PRKCD in human monocyte-derived macrophages during *Mtb* infection. Asterisks are defining significance compared to the control group as: *$p < 0.05$, **$p < 0.01$.

Table S1.

Gene	Accession number	Forward Primer (5'-3')	Reverse Primer (5'-3')
**Mouse PKCδ**	M69042	CTGGGTAACTTAACAAGACC	CTGCTAAATAACATGTTCGGTCC
**Mouse PKCε**	M18331	CATCGATCTCTCGGGATCATCG	CGGTTGTCAAATGACAAGGCC
**Mouse PKCη**	M62980	AGCTAGCCGTCTTCCACGAGACGC	GGACGACGCAGGTGCACACTTGG
**Mouse PKCθ**	D11061	AGCTAGCCGTCTTCCACGAGACGC	GGACGACGCAGGTGCACACTTGG
**Mouse caspase 1**	NM_009807	ACAAGGCACGGGACCTATG	TCCCAGTCAGTCCTGGAAATG
**Mouse caspase 11**	NM_009807	AGAGGGCATGGAGTCAGAGA	GCCATGAGACATTAGCACCA
**Mouse NLRP3**	NM_145827	ATTACCCGCCCGAGAAAGG	TCGCAGCAAAGATCCACACAG
**Mouse AIM2**	NM_001013779	GTCACCAGTTCCTCAGTTGTG	CACCTCCATTGTCCCTGTTTTAT
**Mouse IL-1β**	NM_008361	GCTTCAGGCAGGCAGTATC	AGGATGGGCTCTTCTTCAAAG
**Mouse IL-18**	NM_008360	ACTTTGGCCGACTTCACTGT	GGGTTCACTGGCACTTTGAT
**Mouse GM-CSF**	XM_006532127	ACCACCTATGCGGATTTCAT	TCATTACGCAGGCACAAAAC
**Mouse CSF2RA**	NM_009970	ACGTGGCGCGATGCAT	ACTTGTCACTGCTGGGGAGTG
**Mouse iNOS**	NM_011198	AGCCCTCACCTACTTCCTG	CAATCTCTGCCTATCCGTCTC
**Human PRKCD**	L07860	CACCATCTTCCAGAAAGAACG	CTTGCCATAGGTCCCGTTGTTG
